# Rheumatism: Its Nature, Its Pathology, and Its Successful Treatment

**Published:** 1887-04

**Authors:** 


**Rheumatism: Its Nature, its Pathology, and its Successful Treatment.** By T. J. MACLAGAN, M. D. Octavo, 285 pp., illustrated. Supplied only to subscribers for "Wood's Library of Standard Medical Authors," for 1886. New York: William Wood & Co.

Is there a doctor in the United States who has not heard of Wood's Library? If there is, we would like to inform him that for the small sum of $15 he can get twelve volumes which will contain more valuable reading matter than he can obtain by three times the same expended in individual books. Eichorst's four volumes on "Practical Medicine," for instance, furnish upon the whole the best treatise on practical medicines now extant. Indeed, of the whole twelve volumes for the year 1886, we doubt if there is one which has not been gladly received and heartily appreciated by the subscribers. Certainly from ourselves they have received nothing but favorable mention during the year past. Wood's reputation ensures a continuance of the high quality and value of the library series, and we advise all our readers to send in their subscription for the present year.

